# Initiation and Propagation of Vascular Calcification Is Regulated by a Concert of Platelet- and Smooth Muscle Cell-Derived Extracellular Vesicles

**DOI:** 10.3389/fcvm.2018.00036

**Published:** 2018-04-06

**Authors:** Leon J. Schurgers, Asim C. Akbulut, Dawid M. Kaczor, Maurice Halder, Rory R. Koenen, Rafael Kramann

**Affiliations:** ^1^Department of Biochemistry, Cardiovascular Research Institute Maastricht, Maastricht, Netherlands; ^2^Division of Nephrology, RWTH Aachen University, Aachen, Germany

**Keywords:** extracellular vesicles, vascular smooth muscle cells, perivascular mesenchymal stem cells, vascular calcification, platelets, phenotypic switching

## Abstract

The ageing population continues to suffer from its primary killer, cardiovascular disease (CVD). Despite recent advances in interventional medicinal and surgical therapies towards the end of the 20th century, the epidemic of cardiovascular disease has not been halted. Yet, rather than receding globally, the burden of CVD has risen to become a top cause of morbidity and mortality worldwide. Most CVD arises from thrombotic rupture of an atherosclerotic plaque, the pathologic thickening of coronary and carotid artery segments and subsequent distal ischemia in heart or brain. In fact, one-fifth of deaths are directly attributable to thrombotic rupture of a vulnerable plaque. Atherosclerotic lesion formation is caused by a concert of interactions between circulating leukocytes and platelets, interacting with the endothelial barrier, signalling into the arterial wall by the release of cytokines and extracellular vesicles (EVs). Both platelet- and cell-derived EVs represent a novel mechanism of cellular communication, particularly by the transport and transfer of cargo and by reprogramming of the recipient cell. These interactions result in phenotypic switching of vascular smooth muscle cells (VSMCs) causing migration and proliferation, and subsequent secretion of EVs. Loss of VSMCs attracts perivascular Mesenchymal Stem Cells (MSCs) from the adventitia, which are a source of VSMCs and contribute to repair after vascular injury. However, continuous stress stimuli eventually switch phenotype of cells into osteochondrogenic VSMCs facilitating vascular calcification. Although Virchow’s triad is over 100 years old, it is a reality that is accurate today. It can be briefly summarised as changes in the composition of blood (platelet EVs), alterations in the vessel wall (VSMC phenotypic switching, MSC infiltration and EV release) and disruption of blood flow (atherothrombosis). In this paper, we review the latest relevant advances in the identification of extracellular vesicle pathways as well as VSMCs and pericyte/MSC phenotypic switching, underlying vascular calcification.

## Extracellular Vesicles

Extracellular vesicles (EVs) are currently considered as important physiological players. Their secretion represents a universally active cellular function in all living organisms from bacteria to humans (1). EVs are highly heterogeneous structures that differ in size, biochemical content and mode of secretion. Current nomenclature distinguishes three populations of EVs: (1) exosomes (30–100 nm), which originate when multivesicular bodies fuse with the plasma membrane, (2) microvesicles/ectosomes (100–1,000 nm), which are generated by budding of the plasma membrane and (3) apoptotic bodies (>1,000 nm), which are formed in the process of programmed cell death (2–5). Each family is composed of small phospholipid membrane-enclosed entities released spontaneously, or, in response to cell activation or apoptosis (6). EV release is stimulated via multiple physiological and pathological conditions, making them potential diagnostic biomarkers for monitoring various diseases (7). Their presence has been detected in a number of bodily fluids from healthy individuals, such as peripheral blood, urine, saliva and synovial fluid to name a few (1, 8, 9). In terms of pathological states, first breakthroughs in the field of EV research were made in oncology and immunology, yet today cardiovascular disease represents one of the most intensively studied and rapidly developing areas of the extracellular vesicle field (4). The number of circulating EV levels has been shown to be associated with various cardiovascular and metabolic disorders, including atherosclerosis and diabetes mellitus (8). In the vasculature, EVs are released from platelets, endothelial cells, smooth muscle cells, erythrocytes and leukocytes (6, 7).

### Platelet-Derived Extracellular Vesicles

EVs from platelets were first described in 1967 by Peter Wolf from human blood samples, where they were originally referred to as “platelet dust” (10). Further studies demonstrated that EVs are released when platelets attach to the vessel wall (11). Later it was reported that platelet-EVs are composed of two different types: exosomes and microvesicles (12). Since then platelet EVs have been shown to be involved in several processes in the human body, such as coagulation and atherosclerosis (13). Given that EVs express phospholipids on their surface, they are capable of binding (activated) coagulation factors. Interestingly, their coagulation activity is 50–100 times higher compared to activated platelets (14). In fact, a genetic disorder that is associated with deficient EV formation by platelets leads to bleeding (15). This suggests that promotion of coagulation by EVs is an important physiological mechanism (6).

On the other hand, platelet EVs are known to accumulate in platelet concentrates (16). Transfusion of platelet concentrates is associated with adverse reactions in the recipient, more often than any other blood-derived product (17). This might be explained by the fact that platelet EVs are rich in inflammatory molecules (e.g., CD40L), so they can adhere to leukocytes by CD62P – PSGL-1 interactions and transport pro-inflammatory signals. Several studies have demonstrated that platelet EVs isolated from platelet concentrates can modulate the phenotype and activities of leukocytes and vascular cells (see below). Platelet EVs retain many properties of their parent cells, such as the presence of surface specific antigens (Table 1), the ability to deposit chemokines to the vessel wall and to confer inflammatory signals to distal sites (28). In human blood, platelet-derived EVs are the most abundant population of EVs, despite the fact that erythrocytes are about 30 times more numerous than platelets (29). It has been estimated that in circulation 70–90% of all EVs are derived from platelets, 10% originate from granulocytes and only 5% come from endothelial cells, red blood cells and monocytes (1). The number of circulating platelet EVs is also influenced by cardiovascular medication. Antiplatelet agents, antihypertensive agents and cholesterol-lowering drugs inhibit EV release from platelets (30, 31). However, in studies on patients treated with statins, consensus on the number of circulating platelet EVs has not been established (30, 32). Of note, it has been proposed that the majority of the circulating CD41-positive EVs actually originate from megakaryocytes, rather than from platelets (33). In addition, platelet-derived EVs might be distinguished from their megakaryocyte-derived counterparts through the detection of surface molecules such as CD62P, LAMP-1, CLEC-2 and GPVI.

**Table 1:** Surface markers found on platelet EV.

**Cluster of differentiation (CD) or abbreviation**	**Trivial or full name**	**Reference**
CD9	Tetraspanin-29	(18)
CD29	Integrin β1	(19)
CD31	PECAM-1	(19)
CD36	Platelet GPIV	(20)
CD42a	Platelet GPIX	(21)
CD42b	Platelet GPIbα	(19, 22)
CD63	Tetraspanin-30	(19)
CD59	Membrane attack complex inhibition factor	(20)
CD61	Integrin beta 3	(22)
CD154	CD40 Ligand	(23, 24)
CD184	CXCR4	(23)
PAR-1	Protease-activated receptor-1	(23)
CD321	Junctional adhesionmolecule-A	(25)
TSP-1	Thrombospondin-1	(21)
VN	Vitronectin	(26)
VWF	Von Willebrand Factor	(27)

### Vascular Inflammatory Functions of Platelet-Derived EVs

Besides the involvement of platelet-derived EVs in the coagulation process, evidence also points towards a role in immune- and inflammation-related processes. For example, platelet-derived EVs have been shown to influence vascular cells (endothelial cells and smooth muscle cells) and leukocytes, thereby changing their phenotype and function. EVs are considered to play an important role in cell-cell communication, their membrane-enclosed content, small size and repertoire of surface receptors facilitate long distance transport within bodily fluids (5, 8, 34). EVs can influence target cells by providing ligands which augment the secretion of growth factors or cytokines, transfer of cell adhesion molecules or reprogram target cells through their genetic make up (1, 28, 29). When isolated platelet EVs are incubated with monocytes, platelet EVs readily bind to monocytes and phagocytic uptake of platelet EVs can be observed over time (35). In chemotaxis assays, monocytic cells are actively attracted by platelet EVs, an effect that can be blocked by antibodies against CCL5. Prolonged incubation of monocytes with platelet EVs results in a notable change of surface marker expression, indicating a polarisation of the monocytes to M2-type macrophages (35). Finally, platelet EVs were found to induce the secretion of TNFα from monocytic cells more strongly than platelets, whereas incubation with platelets led to a robust release of GM-CSF (35). Similar studies have demonstrated that platelet EVs induce the differentiation of macrophages into dendritic cells (20) and that platelet EVs are even able to “reprogram” the gene expression profile and function of macrophages (36). Although platelet EVs are present both in diseased patients and healthy subjects, increased levels have been associated with various pathological disorders, such as atherosclerosis and diabetes mellitus (Table 2).

**Table 2:** Cardiovascular/metabolic diseases associated with increased platelet-EV levels.

**Disorder**	**Reference**
Hypercholesterolemia and subclinical atherosclerosis	(37)
Coronary calcification	(38)
Carotid atherosclerosis	(39)
Coronary heart disease	(40)
Acute coronary syndrome	(41–44)
Peripheral arterial disease	(45–48)
Hypertension	(49, 50)
Venous Thrombo-embolism	(51, 52)
Stroke	(53–55)
Diabetes mellitus	(56–59)
Metabolic syndrome and obesity	(60–63)

Table dapted from Aatonen et al. (64) and Ridger et al. (65

Pathological remodelling of the vasculature involves an intricate and dynamic interaction between blood cells (platelets, leukocytes), vascular cells (endothelial cells, smooth muscle cells and adventitial cells) and their direct microenvironment (66). EV-mediated signalling between hematological cells and vascular cells is also of importance in this process. Elevation of platelet EVs in cardiovascular disease appears to be a common process, their interaction with the vascular endothelium has been an area of high interest. However, current knowledge in this field is still very limited and our understanding relies mainly on *in vitro* experiments. The importance of leukocyte-endothelium signalling in pathophysiological conditions of the vasculature is already well known. More recently the role of platelet EVs in these processes has been demonstrated. *In vitro* experimentation has shown that shear stress-activated platelet EVs facilitate the interaction between monocytes and endothelial cells. This is facilitated by increases in inflammatory cytokine levels and cell adhesion molecules on both cell types (67). Another study indicated that platelet EVs contain bioactive lipids (e.g., arachidonic acid), that stimulate ICAM-1 expression in HUVECs, leading to enhanced monocyte-endothelial interactions (68). Platelet EVs may also activate endothelial cells and leukocytes, more specifically, neutrophils by surface molecules CD41 and CD62P, further demonstrating their importance in modulating inflammation (69). It has been also shown that platelet EVs can deposit inflammatory molecules such as CCL5 (RANTES) during rolling interactions over endothelial cell monolayers, facilitating the subsequent recruitment of monocytes (25). In this study, rolling interactions depended on P-selectin and GPIb, while transfer of CCL5 was dependent on integrin αIIbβ3 and junctional adhesion molecule A (25).

Interestingly, more than 700 miRNAs have been found to be stored in platelets (70). Platelets also can contain the functional miRNA processing machinery required for the processing of miRNA precursors (71). Moreover, evidence is accumulating that platelets and platelet-EVs can horizontally transfer nucleic acids to endothelial cells (72, 73). Platelet EVs isolated from activated platelets contain significant amounts of miRNA, further to this, functional transfer of miRNA from platelets to endothelial cells was found to occur through vesicle formation (74, 75). Uptake of platelet EV-associated miRNA results in modulation of endothelial target gene expression demonstrated by a downregulation of ICAM-1 (72).

Besides the endothelial lining of the vessel wall, VSMCs can also be influenced by platelet EVs. Platelets can adhere directly to VSMCs, facilitated by the interaction of CX_3_CR1 on platelets with CX_3_CL1 on VSMCs (76). Although platelets do not make contact with VSMCs under healthy conditions, such encounters might occur after vascular damage, increased endothelial permeability or through intraplaque haemorrhage. Upon endothelial denudation, platelets in a thrombus might release EVs that then come in contact with VSMCs. Increased permeability of the endothelial lining might permit the passage of circulating EVs and during intraplaque haemorrhage, EVs might be formed due to platelet contact with the highly thrombogenic plaque interior. On the other hand, a study investigating EVs isolated from atherosclerotic plaques did not observe a significant fraction of platelet-EVs within plaque (77). It is possible that platelet-EVs lose their surface markers by enzymatic shedding after activation (78), that platelet-EVs are rapidly phagocytosed by macrophages (35, 36), or that platelet-EVs indeed constitute only a minor fraction of the total EV content within a plaque. Nevertheless, a potential influence of platelet EVs on the behaviour and phenotype of VSMCs was investigated in our recent work. Binding studies using CFSE-labelled platelet EVs and VSMCs revealed that the platelet EV–VSMC interaction is metal ion-dependent and that αIIbβ3 on platelet EV is the primary receptor that mediates interactions with VSMCs (79). Platelet EVs induce migration and proliferation of VSMCs in a CXCL4-dependent manner. Prolonged incubation of VSMCs with platelet EVs results in an increased adhesiveness for THP1 monocytic cells under flow conditions and an increase in interleukin 6 production, indicating that platelet EVs have pro-inflammatory effects on VSMCs. Interestingly, the incubation of cultured VSMCs with platelet EVs led to a phenotypic switch towards a synthetic phenotype, as evidenced by morphological changes and a reduced expression of the contractile marker calponin (79). Although direct contact of VSMCs with platelet EVs leads to changes in proliferation, migration, marker expression and phenotype of VSMCs, the possibility exists that platelet EVs might alter the behavior of surrounding cells e.g., the endothelium, thereby indirectly affecting VSMCs by released factors or the transfer of endothelial EVs, analogous to what has been observed during abnormal shear stress (80).

Taken together, platelet EVs influence both phenotype and behaviour of leukocytes and vascular cells, thus are important initiators and propagators in vascular remodelling and downstream processes, such as calcification.

### Initiation and Propagation of Vascular Calcification Is Regulated by Vascular Smooth Muscle Cell Function

Vascular smooth muscle cells (VSMCs) are the most abundant cell source of the vasculature. Unlike most cells, VSMCs arise from several lineages (81). They are critical to maintaining structure and function of the vascular system (82). Their role is central to vessel dilation and constriction as well as vessel remodelling. VSMCs produce components of the vascular extracellular matrix (ECM), therefore altering the composition of connective tissue and can increase the number of VSMCs present in the vasculature by proliferating. VSMCs are commonly considered to be heterogeneous, having either contractile or non-contractile (synthetic) properties (83). This heterogeneity is present in both developing and adult vasculature and is the most defining feature of VSMCs. It has been hypothesised that the different characteristics and functions of VSMCs originate from early developmental cues, as well as spatiotemporal gene regulation of differentiation markers (84).

While in a contractile state, VSMCs contract and relax to enable blood flow around the body. In this contractile state, they express highly VSMC-specific markers for contractility such as SM-αA, calponin and SM22α. These cells have low motility, hence decreased cellular migration is observed, as well as decreased levels of proliferation and a reduced production of extracellular matrices. This enables the blood vessels to maintain elasticity allowing proper function and delivery of blood supply to the anatomy. When synthetic, VSMCs exhibit a marked decrease in expression for VSMC-specific contractility markers, but express more highly markers for matrix metalloproteinase, collagenase, osteopontin and an increase in production of EVs. Phenotype switching enables VSMCs to maintain blood flow as well as support the vascular niche. During vessel repair, migration and proliferation of VSMCs is necessary. Additionally, increases in expression of growth factors such as PDGF, TGF and VEGF, as well as an increased production of ECM is required to reconstruct vasculature following injury.

### Vascular Smooth Muscle Cell Phenotypic Switching

Terminal differentiation of VSMCs is not a definitive end and it is possible to switch between phenotypes depending on the demand of the vascular niche. Contractile VSMCs are generally referred to as quiescent differentiated cells, whereas the synthetic state is associated with plasticity and appropriately referred to by some as dedifferentiated VSMCs. Several pathologies of the vasculature are associated with VSMC phenotype switching such as atherosclerosis, restenosis, aneurysm and calcification (85–87). Vascular calcification, the deposition of hydroxyapatite crystals along the vessel, decreases vessel flexibility, impairs proper blood flow and is associated with cardiovascular disease mortality (88).

While culture conditions for *in vitro* maintenance of human VSMCs in either synthetic or contractile phenotypes have been identified, the precise mechanisms that enable VSMC phenotype switching remains unknown (89). Literature has sparse number of papers implicating miRNAs and proteins in either maintenance or inducing VSMC phenotype switch (90). Furthermore, several identified miRNA and proteins have been eluded to modulation of vascular pathologies associated with VSMC phenotype switching or a dysregulation in phenotype switching (90). During pathological phenotypic switching, VSMCs can adapt an osteogenic, chondrogenic or inflammatory phenotype (91–93).

Adding further interference to the quandary of VSMC phenotype switching, wall resident adventitial progenitor cells have been recently implicated as vessel wall regulators (94, 95). Adventitial progenitors have been found to differentiate into osteoblasts, chondrocytes, adipocytes, macrophage as well as VSMCs. This has led to the hypothesis that adventitial progenitor cells are master regulators of the vascular niche. When progenitor dysfunction occurs, differentiation of VSMCs to an osteoblastic, chondrogenic or macrophage-like capacity are formed. This is yet to be fully appreciated.

### VSMC-Derived Extracellular Vesicles

In 1967, Anderson first used the name “matrix vesicles” in cartilage development and calcification (96). Tanimura and co-workers were the first to report an association between small membrane encapsulated particles, matrix vesicles, and vascular calcification (97). Vesicular structures have been found in both intimal and medial layers and are likely derived from VSMCs. The release of EVs from VSMCs was first described as a rescue mechanism against calcium overloading, in an attempt to prevent apoptosis (98).

Today, it is appreciated by many that the role of EVs released by VSMCs is significant in VSMC phenotype switching and calcification. VSMCs have been found to release a variety of EVs when in either synthetic or osteogenic phenotype. A group of VSMC EVs have been identified and somewhat characterised by tetraspanin markers CD9, CD63 and CD81 (99). Furthermore, EVs share similarity to osteoblast EVs, having calcium binding capacity and osteoblast-like ECM production. Interestingly, calcific conditions *in vitro* increase expression of SMPD3 and subsequent EV genesis. Inhibition of SMPD3 completely ablates generation of EVs and calcification. Additionally, *in vivo* identification of CD63 with calcification of vessels of chronic kidney disease (CKD) patients implicates that SMPD3 is a potential novel therapeutic target to inhibit EV genesis and thus vascular calcification.

Chen and associates recently identified a novel role between VSMCs and EVs in calcification (100). Initially they characterised EVs from four sources, two from cellular-derived EVs the others from matrix vesicles. Curiously, they identified that both populations of cellular-derived EVs possessed the capacity to enhance calcification, however matrix vesicles did not possess this ability, even though all four EV types were uniformly endocytosed by VSMCs. They eluded to novel increases in expression for MEK1, Erk1/2, Nox1 and SOD2 alongside increases in intracellular calcium ion content, EV biogenesis and calcification. All EVs were found to express tetraspanins CD9, CD63 and CD81, however, the proportion of expression differed significantly between the media and cellular EVs. Cellular-derived EVs expressed more significantly for CD63 that strikingly coincides with Kapustin et al. *in vivo* observation of CD63 co-localisation with calcification in calcified CKD vessels (99).

Differences between mineralising and non-mineralising EVs were first revealed by Kirsch et al. within chondrocytes, where they identified high expression of annexin A5 within calcifying EVs (101). The precise function of annexins in calcification has not been fully unravelled (102). Regarding *in vitro* VSMC calcification, annexin A2 has been highlighted in calcium regulation resulting in VSMC EV generation (103). Annexin A2 may bind to fetuin-A on the cell membrane of VSMCs, this is in turn internalised by endocytosis preventing fetuin-A from blocking mineral formation. Increases of annexin A2, annexin A5 and alkaline phosphatase co-localisation are proportional to decreases in fetuin-A expression within *in vitro* VSMC calcification models. Macrophage-derived EV calcification is induced via binding of annexins A2 and A5 to phosphatidylserine (PS) (104). Annexin A5 with S100A9 binding to PS is critical for osteoblast-derived ECM production, interestingly this mechanism also occurs during macrophage production of calcifying EVs. Further interrogation of annexins A2, A5 and A6 in VSMC calcification, phenotype modulation and EV genesis will add to our understanding of the roles of annexins in the cellular context of VSMCs.

Propagation of calcification via vitamin K-antagonist (VKA) treatment led to the identification of matrix Gla-protein (MGP) as a potent calcification inhibitor (105). Twenty years have now passed since MGP inhibitory action was identified, ten years since the mechanism of MGP activation was eluded to by means of serine phosphorylation and gamma-glutamyl carboxylation (91, 106, 107). More recently it has been demonstrated that VSMC-derived EVs contain copious amounts of Gla-proteins from circulation (108). Prothrombin (PT), like MGP, contains a Gla domain. It has been recently demonstrated that production of calcifying VSMC-derived EVs can be inhibited by PT interaction. The Gla domain of PT interacts with the surface of EVs, preventing nucleation sites for calcification (108). This finding has been substantiated by the localisation of PT in calcified regions of the iliac/femoral arteries from a cohort of patients with CKD. Furthermore, circulating levels of PT are reduced in patients with vascular calcification. Accordingly, in absence of MGP, VSMC-derived EVs act to induce calcification, suggesting a potential novel role for inhibition of calcification via PT-EV interactions.

**Figure 1:**
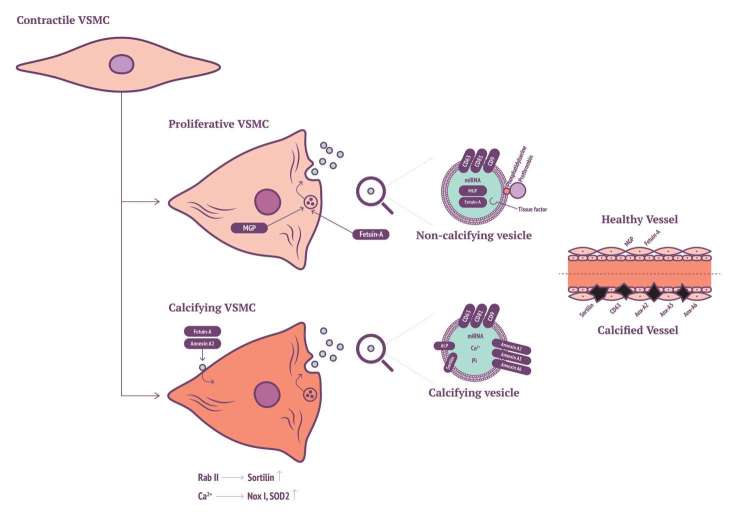
VSMC with Sortilin, MGP and annexin production of calcifying EVs.

Recent findings have implicated a key role for Sortilin in VSMC calcifying EV biogenesis and release. Originally, Sortilin 1 was identified as driving factor in EV-facilitated calcification through Rab11 interaction (109). More recently, it was shown that Sortilin 1 localises in human calcifying vessels (110). In addition, Sortilin 1 plays a role in VSMC calcification *in vitro*. Sortilin 1 contributes to vascular calcification via tissue nonspecific alkaline phosphatase (TNAP) although regulation of TNAP expression within VSMC calcification remains poorly understood. Notably, Sortilin 1 has been identified as a driver in generation of osteoblasts, while TNAP expression was unaffected. This eludes to a potential novel mechanism in the production of VSMC-derived calcifying EVs, which might be a unique mechanism in vascular calcification (Figure 1).

**Figure 2:**
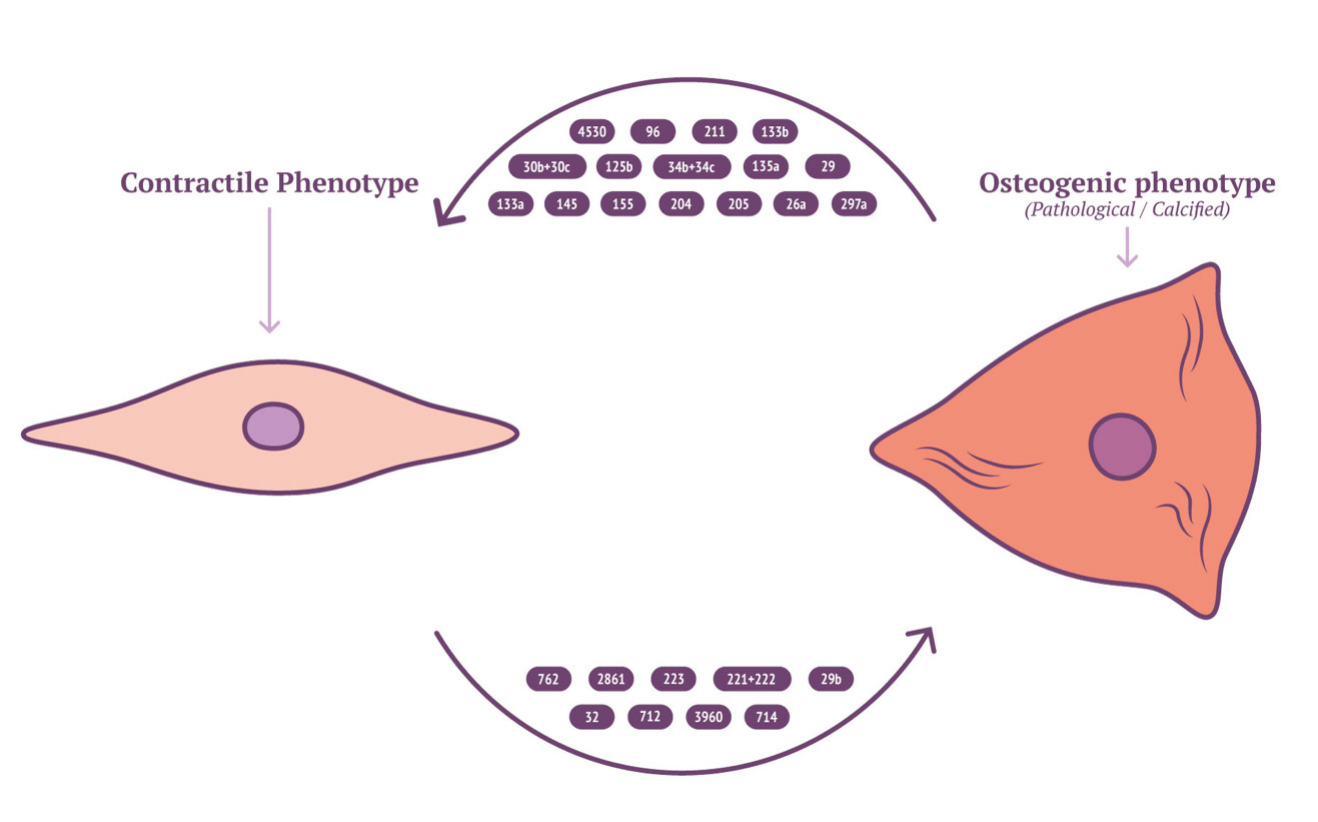
Calcifying miRNAs in VSMC.

Characterisation of EV cargo has been standardised to falling into either protein, RNA or lipid (111). The composition of lipid content in circulating EVs might provide novel insights into mechanisms for VSMC calcification. RNA content of VSMC-derived EVs may be mRNA, miRNA, lncRNA or circRNA, with miRNA being the most characterised in VSMC phenotype modulation and calcification (112). RNA lie within the unique position of being both genotype and phenotype which gives them a role in cellular regulation and pathology (113). Potently a wide array of miRNAs have been implicated by a variety of mechanisms contributing to, or inhibiting the development of vascular calcification (Figure 2 and Table 3). MiRNAs which either induce VSMC to an osteoblast-like pro-calcific phenotype or inhibit associated phenotype and calcification. The specific mechanism of which has been reviewed elsewhere (133, 134). Although the role of miRNAs within vascular calcification is being unravelled, many miRNAs have been identified via RNA screening between normal and calcified VSMCs. The origin of miRNA source in a pathological context remains elusive and requires further investigation.

**Table 3:** MiRNA associated with phenotypic switching of VSMC.

**Reference**	**Calcifying miRNA**	**MiRNA Inhibiting Calcification**	**Paper**
(114)	29b	133b, 211	MicroRNAs 29b, 133b and 211 regulate vascular smooth muscle calcification mediated by high phosphorous
(115)	32		MicroRNA-32 promotes calcification in vascular smooth muscle cells: implications as a novel marker for coronary artery calcification
(116)	3960, 2861		Runx2/miR-3960/miR-2861 positive feedback loop is responsible for osteogenic transdifferentiation of vascular smooth muscle cells
(117)		29	MiR-29-mediated elastin down-regulation contributes to inorganic phosphorous-induced osteoblastic differentiation in vascular smooth muscle cells
(118)		125b	MiR-125b regulates calcification of vascular smooth muscle cells
(119)		135a	MiR-135a suppresses calcification in sensence VSMCs by regulating KLF4/STAT3 pathway
(120)		204	MicroRNA-204 regulates vascular smooth muscle cell calcification *in vitro* and *in vivo*
(121)	221 + 222		MiRNA-221 and miRNA-222 synergistically function to promote vascular calcification
(122)		125b, 145, 155	Decreased microRNA is involved in the vascular remodelling abnormalities in chronic kidney disease (CKD)
(123)	762, 714, 712		Micro RNAs that target Ca(2 + transporters are involved in vascular smooth muscle cell calcification
(124)		30b, 30c	Bone morphogenetic protein-2 decreases microRNA-30b and microRNA-30c to promote vascular smooth muscle cell calcification
(125)		96	Down-regulation of miR-96 by bone morphogenetic protein signalling is critical for vascular smooth muscle cell phenotype modulation
(126)		205	MicroRNA-205 regulates the calcification and osteoblastic differentiation of vascular smooth muscle cells
(127)		133a	MiR-133a modulates osteogenic differentiation of vascular smooth muscle cells
(128)		4530, 133b	Differential expression of microRNAs in severely calcified carotid plaques
(129)		26a	MiR-26a regulates vascular smooth muscle cell calcification *in vitro* through targeting CTGF
(130)		297a	MicroRNA-297a regulates vascular calcification by targeting fibroblast growth factor 23
(131)	223		Inorganic phosphate accelerates the migration of vascular smooth muscle cells: Evidence for the involvement of miR-223
(132)		34b + 34c	MicroRNA-34b/c inhibits aldosterone-induced vascular smooth muscle cell calcification via a SATB2/Runx2 pathway

Whether there is cross talk of EVs from different facets of the vascular niche or if there is a dysregulation in VSMC maintenance remains an important question. Understanding the cross talk of EVs between vascular cells might be significant to appreciating the precise mechanisms for vascular calcification. MiR-206, ARF6 and NCX1 have been identified as endothelial cell-released EV content capable of regulating VSMC contractile phenotype (135). Furthermore, given the observed increase of endothelial EV release following injury, it has been noted that endothelial cell EVs from human pulmonary artery not only interacted with pulmonary VSMCs, but interaction induced proliferation and had a seemingly anti-apoptotic effect on VSMCs (136). This suggests a direct pathological consequence of endothelial EV to VSMC crosstalk during and following intimal vessel injury.

## Mesenchymal Stem Cells as Perivascular Progenitors

As described above, VSMCs are known to exhibit elaborate phenotypical plasticity and diversity during normal development, disease, and repair of vascular injury. The current theory of vascular calcification is that upon injury VSMCs dedifferentiate. VSMCs become synthetically active and under stress factors, like elevated calcium and phosphate levels or uremic toxins (e.g., during CKD), switch to a dedifferentiated synthetic phenotype and later become osteoblast like cells (99, 137). Genetic fate tracing strongly indicated mature VSMCs to be a major contributor to the development of atherosclerotic plaque remodeling (138). However, not all elements of plaque development and progression may be indicative as a result of VSMC phenotype switching. The fact, that atherosclerotic human vessels can contain complete trabecular bone with fully formed bone marrow sinusoids even containing hematopoietic cells is difficult to explain without new concepts, such as vascular stem cells. Indeed, the presence of a specialised progenitor population of VSMCs localised in the adventitia of muscular arteries has been suggested by several groups.

The perivascular niche houses pericytes, which are present at intervals along microcapilaries and pericyte-like cells are also located in the adventitia of large arteries. Peault and coworkers were the first to show that many pericytes are MSCs (139, 140). However, it remains elusive whether all pericytes are MSCs. A pericyte is defined as a cell that is completely or partially embedded in the endothelial basement membrane (141). The fact that MSCs are also located in the adventitia of large arteries distant from vasa vasorum and in the endosteal niche of the bone marrow indicates that not all MSCs are pericytes (94, 142). The recent evidence that the perivasculature represents the MSC niche explains why MSCs can be isolated from virtually all organs and tissues.

It was first described over 25 years ago that pericytes have an osteogenic potential (143) and subsequently pericyte-like cells with osteogenic capacity were isolated from VSMCs nodules of human aorta (144). A decade ago it has been reported that Sca1^+^/CD34^+^/PDGFRβ^+^ cells that reside in the adventitia of arteries possess a differentiation capacity towards smooth muscle cells and osteoblasts *in vitro* (145). Interestingly, these progenitors express the Hedgehog receptor Ptc1 as well as most other hedgehog pathway members including Gli1-3 (145). Other groups have reported that perivascular MSCs were also found to be progenitors of white adipocytes (146) follicular dendritic cells (147) and skeletal muscle (148), while also playing a major role in fibrotic response (149–151). Thus, various studies suggest the presence of adventitial MSC-like cells with osteogenic and myogenic potential. However, until recently the involvement of these MSC-like pericytes in cardiovascular disease development remained unclear.

### Role of Perivascular MSC-Like Cells in Vascular Calcification

Using genetic labeling and *in vivo* fate tracing experiments it was recently revealed that the Hedgehog transcriptional activator Gli1 specifically labels perivascular MSC-like cells (94, 142, 151, 152). Gli1^+^ cells reside in the pericyte niche with direct contact to endothelial cells of the microvasculature and in the adventitia of large arteries (94, 151). Gli1^+^ cells possess all criteria that have been used to define a MSCs including surface marker expression, tri-lineage differentiation and plastic adherence (94, 151). Inducible genetic fate tracing, the gold standard technique to dissect cellular hierarchies, indicated that Gli1^+^ cells are a major cellular source of myofibroblast in fibrosis of all major organs such as lung, kidney, liver, heart and bone marrow (142, 151).

The question is however, whether adventitial Gli1^+^ MSC-like cells are involved in acute injury repair and chronic vascular disease progression. Using *in vivo* genetic fate tracing, it was demonstrated that after wire injury of the femoral artery about 50% of newly formed VSMCs were derived from adventitial Gli1^+^ cells (94). Furthermore, FACS isolated adventitial Gli1^+^ MSC could be differentiated into calponin^+^, αSMA^+^, smoothelin^+^ VSMCs in vitro (94). This data indicates that adventitial Gli1^+^ MSC are indeed progenitors of VSMCs.

The role of adventitial Gli1^+^ MSC in vascular calcification was studied in triple transgenic Gli1CreER; tdTomato, ApoEKO mice. Mice were pulsed with tamoxifen in order to genetically tag Gli1^+^ cells by expression of the bright red fluorochrome tdTomato. Mice were subjected to either subtotal nephrectomy and western diet or sham surgery with standard chow. Interestingly, a continuous low frequency migration of Gli1^+^ cells into the media during aging in the sham group was observed. Gli1^+^ cells acquired markers of VSMCs such as α-SMA and calponin suggesting that progenitor cells continuously replace lost VSMCs during aging (94). Importantly, these data indicate a great migration of Gli1^+^ cells into the media and neointima during chronic injury and atherosclerosis. Multiple co-staining experiments indicated that adventitial Gli1^+^ cells first differentiated into contractile VSMCs (α-SMA^+^, calponin^+^) and then underwent a phenotypic switching with loss of contractile VSMC markers and acquisition of synthetic VSMC markers such as Tropomyosin alpha 4 (TPM4) and non-muscle myosin heave chain 2b (nmMHC2b) (94). Importantly, during vascular calcification a high percentage of Gli1^+^-derived cells acquired nuclear expression of the transcription factor Runx2 indicating differentiation into osteoblast-like cells. Imaging experiments after injection of a fluorochrome conjugated bisphosphonate (Osteosense) indicated that calcified areas showed intense accumulation of tdTomato^+^ cells. Importantly, genetic ablation of Gli1^+^ cells in triple transgenic Gli1CreER; iDTR; ApoEKO mice before onset of CKD completely abolished vascular calcification in both the intima and media. Thus, clearly demonstrating that adventitial Gli1^+^ cells are important progenitors of synthetic VSMCs and osteoblast-like cells in the vascular wall. Adventitial Gli1^+^ cells can be considered an important therapeutic target in vascular calcification. Interestingly, we observed a Shh domain in the adventitia of human arteries where endothelial cells of vasa vasorum stained positive for Shh, whereas surrounding cells showed Gli1 expression. Further staining experiments in calcified human arteries showed intense Gli1 expression around calcified intima and media areas.

**Figure 3:**
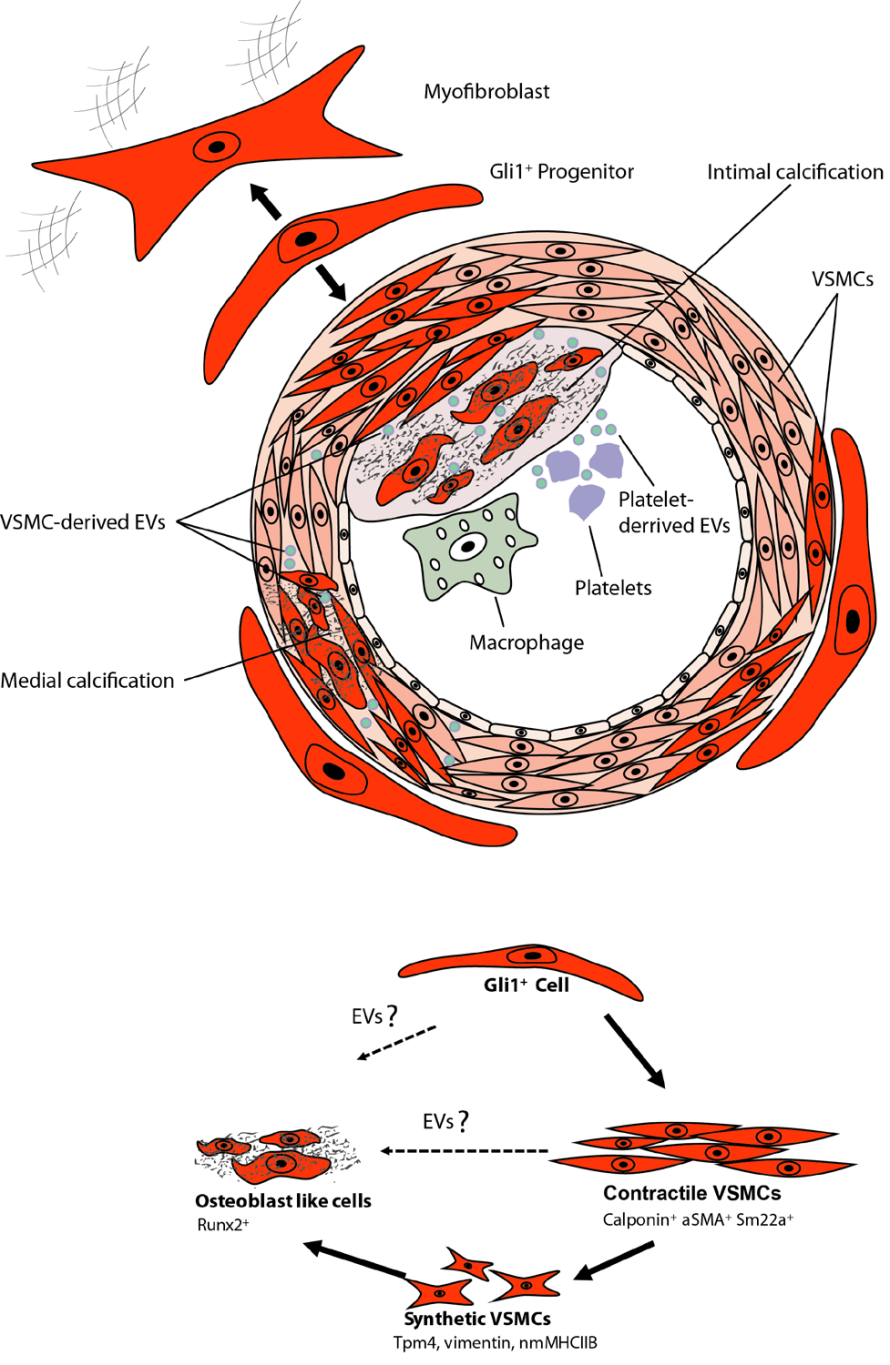
Calcifying EVs and Gli1+ Progentior Cells.

While these data clearly demonstrate an important role of adventitial MSC-like cells in vascular calcification there are still various open questions that need to be answered (Figure 3). It is unclear whether the Gli1^+^ population is a homogenous progenitor population or whether several subpopulations exist. Single-cell qPCR analysis of sorted Gli1^+^ cells for reported markers of adventitial progenitors indicates heterogeneity with three distinct subpopulations (unpublished data). Thus, it will be important to differentiate between these subpopulations. Next, it remains elusive as to whether Gli1-expressing cells of human arteries may also secrete EVs and thereby contribute to vascular calcification. Finally, while our data clearly indicates migration and differentiation of Gli1^+^ cells during acute injury repair and chronic disease progression, the underlying molecular pathways that activate migration and differentiation of the progenitor population remain obscure. It is currently not known whether EVs are involved in migration and differentiation of MSCs. Further, we must await as to whether adventitial Gli1^+^ cells produce EVs or interact with EVs from other cells such as platelets, immune cells and VSMCs. Experiments in myelofibrosis demonstrated that a malignant hematopoietic clone in particular megakaryocytes can activate Gli1^+^ cells to leave their niche partly by CXCL4 release (142). Thus, involvement of platelet and cellular EVs might be an explanation for activation and migration of Gli1^+ ^cells. Further studies are needed to answer whether EVs are involved in Gli1^+^ cell recruitment and differentiation.

## Conclusion

The biology of vascular disease and calcification is complex and still poorly understood with respect to cause and consequence of players and pathways, and thus complexity increases continuously by ongoing elucidation of novel players and pathways. A subset of EVs act as mediators of cell-induced extracellular matrix calcification in the pathogenesis of cardiovascular disease. On the other hand, it has become evident that platelet and cellular vesicles play an important role in cellular communication. Whether such communication from platelet EVs can transpire through endothelial cells to the media and adventitia remains unknown, and so a role for EV crosstalk in calcification remains open with promise.

## Author Contributions

LS wrote the manuscript, supervised writing process and was responsible for the final version; AA wrote the manuscript; DK wrote the manuscript; MH wrote the manuscript; RRK wrote the manuscript; RK wrote the manuscript and was responsible for the final version.

## Conflict of Interest Statement

The authors declare that the research was conducted in the absence of any commercial or financial relationships that could be construed as a potential conflict of interest.
